# The Effect of Multiple Rounds of Mass Drug Administration on the Association between Ocular *Chlamydia trachomatis* Infection and Follicular Trachoma in Preschool-Aged Children

**DOI:** 10.1371/journal.pntd.0002761

**Published:** 2014-04-10

**Authors:** Jennifer S. Lee, Beatriz E. Muñoz, Harran Mkocha, Charlotte A. Gaydos, Thomas C. Quinn, Sheila K. West

**Affiliations:** 1 Dana Center for Preventive Ophthalmology, Wilmer Eye Institute, Johns Hopkins University, Baltimore, Maryland, United States of America; 2 Kongwa Trachoma Project, Kongwa, Tanzania; 3 Division of Infectious Diseases, Department of Medicine, Johns Hopkins University, Baltimore, Maryland, United States of America; 4 Division of Intramural Research, National Institute of Allergy and Infectious Diseases, National Institutes of Health, Bethesda, Maryland, United States of America; University of California San Francisco, United States of America

## Abstract

**Purpose:**

To examine the relationship between ocular *Chlamydia trachomatis* infection and follicular trachoma (TF) in children prior to and following multiple rounds of annual mass drug administration (MDA) with azithromycin.

**Methodology/principal findings:**

Thirty-two communities with endemic trachoma in Kongwa District, Tanzania, were offered annual MDA as part of a district-wide trachoma control program. Presence of ocular *C. trachomatis* infection and TF were assessed in 3,200 randomly sampled children aged five years and younger, who were examined prior to each MDA. Infection was detected using the Amplicor CT/NG assay and TF was identified by clinical examination using the World Health Organization (WHO) simplified grading system. The association between chlamydial infection and TF in children was evaluated at baseline prior to any treatment, and 12 months after each of three annual rounds of mass treatment. Factors associated with infection were examined using generalized estimating equation models.

At baseline, the overall prevalence of chlamydial infection and TF was 22% and 31%, respectively. Among children with clinical signs of TF, the proportion of those with infection was 49% prior to treatment and declined to 30% after three MDAs. The odds of infection positivity among children with clinical signs of TF decreased by 26% (OR 0.74, 95% CI 0.65 to 0.84, *p* = <0.01) with each MDA, after adjusting for age. For children aged under one year, who did not receive treatment, the relationship was unchanged.

**Conclusions/significance:**

The association between ocular *C. trachomatis* infection and TF weakened in children with each MDA, as both infection and clinical disease prevalence declined. However, there was still a significant proportion of TF cases with infection after three rounds of MDA. New strategies are needed to assess this residual infection for optimal treatment distribution.

## Author Summary

Trachoma, which is caused by infection by the bacterium *Chlamydia trachomatis*, is the leading preventable cause of blindness worldwide. Annual mass drug administration with azithromycin is recommended for trachoma control; however, monitoring the impact of azithromycin, which targets *C. trachomatis*, relies on the clinical assessment of follicular trachoma. If the relationship between chlamydial infection and the presence or absence of follicular trachoma were to remain unchanged with each round of treatment, we would be able to predict the level of residual infection, and the need for additional treatment, from the prevalence of follicular trachoma. In this study, we examined the association between infection and presence or absence of follicular trachoma in children prior to and following multiple rounds of treatment. Findings suggest that with increasing rounds of treatment, the prevalence of infection declines in children both with and without signs of follicular trachoma. Newer strategies, including tests that can rapidly detect infection under field conditions, may be needed to assess residual infection in treated communities.

## Introduction

Trachoma, the most common preventable cause of blindness in the world [1], [2], is caused by repeated and/or prolonged episodes of ocular infection by the bacterium *Chlamydia trachomatis*. The disease disproportionately affects individuals living in rural and resource-poor settings, and children are the primary carriers of ocular *C. trachomatis* infection and active disease [3]. We and others have previously shown that clinical disease persists longer than infection in children such that in any prevalence survey, a significant proportion of children will have active trachoma without demonstrable signs of *C. trachomatis* infection [4], [5], [6].

Trachoma control programs rely on a package of interventions developed by the World Health Organization (WHO) that comprises Surgery for trichiasis, Antibiotics for reducing infection, Facial cleanliness, and Environmental improvement, which is referred to as the SAFE strategy [7], [8]. Annual mass drug administration (MDA) with single-dose azithromycin is recommended for trachoma control in communities in which the prevalence of follicular trachoma (TF) is 10% or greater in children aged 1–9 years, with the aim of reducing TF prevalence to under 5% [8]. At least three years of MDA are recommended before reassessing the need for further MDA; however, monitoring the impact of antibiotic intervention, which targets *C. trachomatis*, relies on the clinical assessment of TF. If the relationship between chlamydial infection and TF remained constant with each MDA, one should be able to predict the level of residual infection in treated communities, and thus the need for further antibiotics, from the prevalence of TF.

As part of a randomized community trial, we evaluated ocular chlamydial infection and TF in communities located in the Kongwa District of Tanzania, where the prevalence of active trachoma ranged from 20% to 50% prior to any treatment [9], [10]. These communities have since undergone several years of annual MDA with azithromycin. The aim of the study described here was to examine the relationship between ocular *C. trachomatis* infection and TF in these communities prior to and following multiple rounds of MDA.

## Methods

### Communities and index children

A total of 32 communities in Kongwa District, Tanzania, were selected for the community trial, which included annual mass treatment [9], [10]. A census of all households in the communities enrolled in the study was conducted to collect basic demographic information on each family member. Based on the census list for each community, a sample of 100 index children aged five years and younger was randomly selected for each survey, for a total of approximately 3,200 children examined prior to each round of mass treatment [9], [10].

Individual written informed consent from each child's parent or guardian was obtained prior to ocular examination. All study protocols and procedures were approved by the institutional review boards at the Johns Hopkins University and the National Institute for Medical Research in Tanzania. The community trial is registered on ClinicalTrials.gov under NCT00792922.

### Data collection

Ocular examinations and assessments were conducted prior to any treatment, and 12 months after each of three annual rounds of mass treatment. Standardized graders examined and graded both eyelids of each index child using the WHO simplified grading system [11], which assesses the presence or absence of TF, intense trachoma (TI), trachomatous scarring (TS), trachomatous trichiasis (TT), and corneal opacity (CO). For this study, the relevant signs of trachoma were TF and TI. For quality control purposes, a subset of the index children in each community was randomly selected to have ocular photographs taken using a Nikon D-series camera, and these photographs were graded by a single grader. No drift in grading over time was detected, and kappa coefficients for intergrader agreement were above 0.7 for assessment of TF at all times [12].

Ocular swabs were collected from the left upper eyelid of each index child. A Dacron swab (Fisher HealthCare, Houston, TX) was rotated and swiped across the upper conjunctiva three times and placed dry in a vial. Vials were placed in a cooler in the field, transferred to a refrigerator at the end of the day, and temporarily stored there until shipped within 30 days of collection to the International Chlamydia Laboratory at the Johns Hopkins University. Field protocols to avoid contamination of specimens were strictly followed, including changing gloves between examinations and collecting “air” controls to monitor field contamination. No evidence of field contamination was identified during the entire study [12].

All ocular specimens were processed for detection of *C. trachomatis* in the laboratory using the AMPLICOR CT/NG test (Roche Molecular Diagnostics, Indianapolis, IN) according to manufacturer's specifications. Each ocular swab was eluted by vortexing in lysis buffer in polypropylene tubes, after which diluent was added. Using a known positive sample, two positive and two negative processing controls were run with each batch of specimens. After the hybridization reaction, the optical density (OD) for each specimen was measured. Samples with ODs of 0.8 or greater were recorded as positive for *C. trachomatis* and evidence for infection; samples with ODs less than 0.2 were recorded as negative, while samples with ODs between 0.2 and less than 0.8 were considered equivocal. Samples with equivocal results were retested in duplicate; samples that retested equivocal or repeated as negative on two occasions were considered negative. Less than 0.1% of specimens were equivocal. Further details on quality assurance are discussed elsewhere [10], [13].

### Treatment

After ocular examinations and assessments were completed, treatment was offered to all community residents, and comprised a single dose of oral azithromycin at 20 mg/kg for adults and children over six months of age. A 1% topical tetracycline eye ointment was provided for children under six months of age with instructions to apply twice daily for four to six weeks. Antibiotic coverage exceeded 80% for children aged under 10 years in all communities at each MDA [12]. Treatment coverage at baseline, one year, and two years in children aged 0–5 years was 94% (SD 5.4%), 90% (SD 5.5%), and 90% (SD 4.9%), respectively. Compliance at six weeks with topical tetracycline among infants was not measured. Data are reported as number of rounds of community treatment offered, and are not reflective of the specific number of treatments given to each child.

### Statistical analysis

All statistical analyses were conducted using SAS 9.2 software (SAS Institute Inc., Cary, NC). Contingency table analysis was used to examine the relationship at the individual level between infection and TF (with or without TI) and TI alone (with no sign of TF), stratified by child's age, gender, and the community disease prevalence prior to the most recent round of treatment. The association between ocular chlamydial infection and TF and TI alone (with no sign of TF) was evaluated at baseline prior to any treatment, and 12 months after each of three rounds of MDA. There was no statistically significant difference by treatment arm in the original trial [12], so all children from these communities were combined for these analyses. Among children with clinical signs of TF, factors associated with the presence of infection were examined using single and multiple logistic regression modeling, and a generalized estimating equation was employed to account for clustering of infection within communities. The adjusted model included age as well as number of rounds of MDA in the community.

## Results

At baseline, there was an average of 1,457 residents in each of the 32 communities included in the study (Table 1). Overall, 76% of households reported having a primary water source during the dry season that was over 30 minutes away, and 65% of households were observed to have a latrine. Heads of household reported having completed an average of 3.3 years of formal education.

**Table 1:** Mean characteristics of 32 communities at baseline.

Characteristic	Mean ± SD
Number of residents	1457±429
Education level of head of household (years)	3.3±0.9
Households with no nearby water source (over 30 minutes away)	76%±24%
Households with latrine	65%±17%

**Figure 1:**
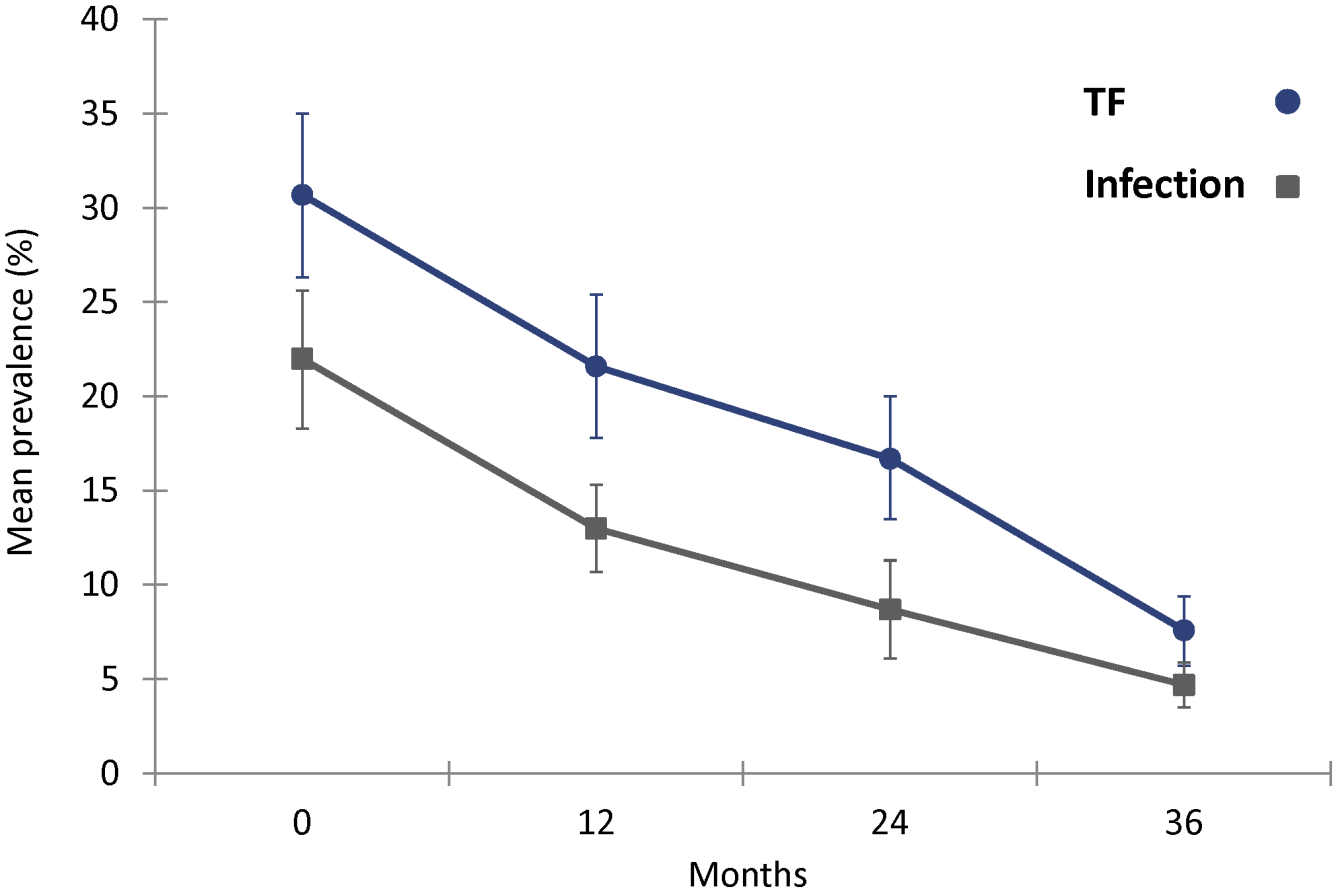
Mean prevalence (and 95% confidence intervals) of *C. trachomatis* infection and TF in 32 communities over time.

The baseline mean prevalence of ocular chlamydial infection was 22%, and the baseline mean prevalence of TF was 31% (Figure 1) [12]. After each successive round of MDA, the overall prevalence of chlamydial infection declined, from 22% at baseline to 5% at 36 months, for a reduction of 79%. The prevalence of TF declined from 31% to 8%, for a reduction of 75%. At baseline, the prevalence of TI alone (without TF) was 1.4%, and this remained low over time; at 36 months, the prevalence of TI alone was 0.5%.

Among children with no signs of active trachoma, 9.0% had infection at baseline prior to administration of any treatment (Table 2). After three rounds of MDA, the proportion of children with infection among those with no signs of active trachoma declined to 2.5%, for a reduction in infection of 72% (*p* = <0.001). Among children with TF, 49% had infection at baseline and 30% had infection after three rounds of MDA, for a reduction in infection of 40% (*p* = <0.001). Among children with TI alone (without TF), 49% had infection at baseline and 35% had infection after three rounds of MDA, for a reduction in infection of 28% (*p* = 0.186).

**Table 2:** Proportion of infection by active disease status and number of MDAs in children aged 5 years and under.

	Disease status								
	Without signs of active trachoma (TF or TI)			Signs of active trachoma					
Number of MDAs				TF (with or without TI)			TI alone		
	N	Proportion infected (%)	p-value	N	Proportion infected (%)	p-value	N	Proportion infected (%)	p-value
0	2114	9.0	<0.001	962	48.9	<0.001	45	48.9	0.186
1	2477	6.1		689	36.9		23	43.5	
2	2631	2.8		535	36.3		32	34.4	
3	2899	2.5		237	29.5		17	35.3	

At baseline, infection increased with increasing age and with community disease prevalence of over 20%, in both children with TF and children with no signs of trachoma (Table 3). There were no significant differences in infection by gender.

**Table 3:** Relationship between trachoma status and infection in children by demographic characteristics at baseline.

	Without signs of active trachoma			TF (with or without TI)		
Characteristic	N	% PCR+	p-value	N	% PCR+	p-value
Age						
<1	486	6.8	**0.001** *	52	42.3	**<0.001** *
1–3	1011	8.2		550	42.7	
4–5	617	12.2		360	59.2	
Gender						
Males	1083	8.3	0.234	485	48.0	0.610
Females	1031	9.8		477	49.7	
Community trachoma prevalence						
20% or under	475	6.3	**0.019**	78	25.6	**<0.001**
Over 20%	1639	9.8		884	50.9	

*Test for trend with age as continuous variable.

In unadjusted analyses, the odds of infection in children with TF decreased with each MDA (OR 0.74, 95% CI 0.65 to 0.85, *p* = <0.001) (Table 4). In the age-adjusted model, the odds of infection in children who had TF significantly decreased with each MDA round (OR 0.74, 95% CI 0.65 to 0.84, *p* = <0.001) (Table 4). For example, if we take a child aged two years, the model suggests that at baseline, the conditional probability of infection given TF is about 0.40; after two rounds of MDA, the probability of infection given TF is 0.26. Based on our model, after four rounds of MDA, the conditional probability of infection given TF is 0.16. An additional analysis was conducted to assess whether the relationship between clinical signs of TF, *C. trachomatis* infection, and number of MDAs changed depending on age. Stratified analysis was performed for children aged 0–1 year and children aged 2–5 years (Table 5). Among children aged 0–1 year with TF, the odds of infection were unchanged with each MDA (OR 0.93, 95% CI 0.77 to 1.12, *p* = 0.425). Among children aged 2–5 years with TF, the odds of infection significantly decreased with each MDA (OR 0.71, 95% CI 0.61 to 0.83, *p* = <0.001) (Table 5).

**Table 4:** Odds ratios for factors associated with *C. trachomatis* infection in children with clinical signs of TF.

	Crude			Adjusted*		
Variable	Odds ratio	95% CI	p-value	Odds ratio	95% CI	p-value
Number of community MDAs	0.74	0.65–0.85	<0.001	0.74	0.65–0.84	<0.001
**Age**						
<1 year**	1.33	0.95–1.96	0.10	1.26	0.88–1.84	0.207
3–5 years**	1.52	1.30–1.79	<0.001	1.54	1.31–1.82	<.001

*Adjusted model included variables for number of community MDAs and age.

** Reference age group for this categorical variable is age 1-<3 years.

**Table 5:** Odds ratios of infection in children with TF in two specific age groups, with each round of MDA.

	Odds ratio		
Age	OR	95% CI	p-value
0–1	0.93	0.77–1.12	0.425
2–5	0.71	0.61–0.83	<0.001

## Discussion

This study indicated that the association between ocular *C. trachomatis* infection and trachoma in children decreased with each successive round of mass treatment, as the overall prevalence of chlamydial infection and TF declined in the communities treated. In our statistical model, the odds of infection in children with TF were reduced by 26% with each round of MDA.

We and others have previously evaluated the cross-sectional association between ocular chlamydial infection and active trachoma at both the community level [6], [14], [15] and individual level [5], [16], [17], [18]. Most have found low levels of infection in individuals without clinical disease and higher levels of infection in those with clinical disease, which we also observed in this study. Higher levels of infection have been generally observed in individuals with TI alone (without TF) than those with TF, and we observed this same trend, though we had few TI cases in our sample.

Keenan et al. [14] observed a reduced association between active trachoma and chlamydial infection following two to three years of biannual treatment in hyperendemic areas of Ethiopia, though the association was studied at the village level and not at the individual level, so it is not possible to evaluate whether the decline was among children who had TF, as we did here. We also observed a decline in the proportion of children with infection who did not have signs of trachoma, which may reflect a decline in pre-clinical disease as trachoma declines with each successive round of MDA.

While TF and infection declined following each round of MDA, we also observed a growing discrepancy between infection and TF, reflecting an increasing proportion of children with TF and no *C. trachomatis* infection. There are several factors that potentially explain the presence of TF in the absence of *C. trachomatis* infection. There may have been cases of ocular inflammation that were recorded as “TF” when in fact the cases were caused by infectious pathogens other than *C. trachomatis* or by mechanical irritants, which has been previously observed [19]; however, the presence of other pathogens was very low in that study and could not entirely explain the cases of TF without chlamydial infection that were observed. Additionally, individuals who live in endemic settings and are continually exposed to *C. trachomatis* may exhibit persistent clinical signs of active trachoma regardless of current infection status [5]. The growing discrepancy may also relate to a likely distribution of resolution periods for follicles following infection, from very fast to very slow. With stable transmission prior to introduction of antibiotics, a prevalence survey would reflect this mix of resolution periods, with a proportion of children with TF and infection as well as a proportion of children with TF and no infection. However, if infection is drastically lowered under antibiotic pressure, then there are fewer opportunities to acquire infection; the result would be a decline in the overall TF prevalence, as was observed here. In addition, a prevalence survey would disproportionately reflect TF cases with longer resolution periods and no infection.

We do not believe that the decline in infection among cases of TF reflects a growing inability to detect organism. While insensitive laboratory tests, such as culture or Giemsa staining, might be sensitive to declining infectious loads following treatment, we used a highly sensitive nucleic acid amplification test. We did not use pooling strategies to detect infection, which can reduce sensitivity due to dilution as infectious load drops. Furthermore, all specimens were processed similarly across the trial with the same number of freeze/thaw cycles, which may also affect test sensitivity.

Finally, it is worth noting that as the prevalence of TF declines in a community, both the prevalence of infection in cases of TF and in those without disease declines, though an increasing proportion of infections overall would be seen in those without disease due to the reduced positive predictive value of TF for infection.

The odds of being infected with *C. trachomatis* increased with age and in those with TF. It has also been observed in previous research that infection and active disease prevalence increases by age among children under age five, and then declines by age once they start school [20]. We studied children aged five years and younger, as that is the age group of children at highest risk of trachoma, as well as being easiest to study in the community. In this population, the Pearson correlation of TF between children aged under five years and those aged under ten years was very high (0.96). Moreover, the odds of infection among children aged 0–<1 year with clinical signs of trachoma remained essentially unchanged with each community MDA. Of note, children aged under one year had not received any treatment at the time of the survey, as they were born after the previous round of MDA was provided, so the lack of change is not surprising. The finding gives further support to the idea that the rounds of MDA were associated with the decline in the association of TF and infection, and not some secular trend that would also affect children aged under one year.

We also note that there is a small risk of infection when transmission is ongoing, and in our study, 2.5% of children without TF had infection after three rounds of MDA. Even though the prevalence of infection in these children is low, in communities with very low TF prevalence, these children can become the primary source of infection. As TF prevalence declines, even fewer infections will be associated with disease, as has been observed [21].

Our model was based on communities with an average trachoma prevalence of 30% at baseline, and with very high treatment coverage rates in children. It is not clear whether findings would differ in communities with a baseline prevalence of trachoma of under 20% or over 50%, or with low treatment coverage at the community level. The association between ocular chlamydial infection and active trachoma may also be impacted by other individual or community factors favoring transmission of infection that were not measured in this study.

These study findings suggest that with increasing rounds of MDA at high coverage, the prevalence of infection decreases in children with TF. In this setting, we can model the change in the relationship between infection and disease, and it seems to hold even when TF prevalence drops below 10%, with the odds of infection in children with TF reduced by about 26% with each round of MDA. Note that some chlamydial infection still exists when TF prevalence is below 10%, indicating the potential ongoing need for MDA. Therefore, it may be worthwhile to investigate newer strategies, including tests that can rapidly detect infection under field conditions [22], [23] or possibly tests for antibodies in young children, to determine whether infection transmission is still occurring, or whether transmission has been interrupted and treatment efforts can be stopped.
